# Stressors and Destressors in Working From Home Based on Context and Physiology From Self-Reports and Smartwatch Measurements: International Observational Study Trial

**DOI:** 10.2196/38562

**Published:** 2022-11-10

**Authors:** Danielle Tump, Nitin Narayan, Vera Verbiest, Sander Hermsen, Annelies Goris, Chui-De Chiu, Ruud Van Stiphout

**Affiliations:** 1 OnePlanet Research Center Stichting imec Nederland Wageningen Netherlands; 2 Department of Psychology The Chinese University of Hong Kong Hong Kong Hong Kong

**Keywords:** stress, telework, wearables, COVID-19, pandemic, remote working, employees, stressors, destressors, remote work, mental health, psychological health, smartphone, digital questionnaire, stress management, occupational health, stress detection, prediction model

## Abstract

**Background:**

The COVID-19 pandemic has greatly boosted working from home as a way of working, which is likely to continue for most companies in the future, either in fully remote or in hybrid form. To manage stress levels in employees working from home, insights into the stressors and destressors in a home office first need to be studied.

**Objective:**

We present an international remote study with employees working from home by making use of state-of-the-art technology (ie, smartwatches and questionnaires through smartphones) first to determine stressors and destressors in people working from home and second to identify smartwatch measurements that could represent these stressors and destressors.

**Methods:**

Employees working from home from 3 regions of the world (the United States, the United Kingdom, and Hong Kong) were asked to wear a smartwatch continuously for 7 days and fill in 5 questionnaires each day and 2 additional questionnaires before and after the measurement week. The entire study was conducted remotely. Univariate statistical analyses comparing variable distributions between low and high stress levels were followed by multivariate analysis using logistic regression, considering multicollinearity by using variance inflation factor (VIF) filtering.

**Results:**

A total of 202 people participated, with 198 (98%) participants finishing the experiment. Stressors found were other people and daily life getting in the way of work (*P*=.05), job intensity (*P*=.01), a history of burnout (*P*=.03), anxiety toward the pandemic (*P*=.04), and environmental noise (*P*=.01). Destressors found were access to sunlight (*P*=.02) and fresh air (*P*<.001) during the workday and going outdoors (*P*<.001), taking breaks (*P*<.001), exercising (*P*<.001), and having social interactions (*P*<.001). The smartwatch measurements positively related to stress were the number of active intensity periods (*P*<.001), the number of highly active intensity periods (*P*=.04), steps (*P*<.001), and the SD in the heart rate (HR; *P*<.001). In a multivariate setting, only a history of burnout (*P*<.001) and family and daily life getting in the way of work (*P*<.001) were positively associated with stress, while self-reports of social activities (*P*<.001) and going outdoors (*P*=.03) were negatively associated with stress. Stress prediction models based on questionnaire data had a similar performance (*F*_1_=0.51) compared to models based on automatic measurable data alone (*F*_1_=0.47).

**Conclusions:**

The results show that there are stressors and destressors when working from home that should be considered when managing stress in employees. Some of these stressors and destressors are (in)directly measurable with unobtrusive sensors, and prediction models based on these data show promising results for the future of automatic stress detection and management.

**Trial Registration:**

Netherlands Trial Register NL9378; https://trialsearch.who.int/Trial2.aspx?TrialID=NL9378

## Introduction

In March 2020, the World Health Organization declared the outbreak of COVID-19 a global pandemic, which lasted until the beginning of 2022 [1]. It forced people to work from home full-time to minimize the spread of the COVID-19 virus. Employees had to quickly adapt their way of working and their personal lives, as not only offices but also schools, sport facilities, and day cares were closed. This new way of working, however, was associated with social deprivation, lack of exercise, home confinement, and additional stress from managing work while taking care of children [2]. Even though some companies have returned to the office full-time, many companies continue to offer hybrid or completely remote work after the pandemic [3-5].

Prior to the pandemic, research into the link between stress and remote employment had yielded conflicting results, with small effect sizes [6-9]. Known mediators of remote work–induced stress were job autonomy, work-life conflict, and work-life balance [7,8]. Working remotely during the pandemic, however, differs radically from prior remote work arrangements, in that it is involuntary; independent of employees’ wishes, personal characteristics, and circumstances; independent of organizational culture; and coinciding with the staying at home of other members of the household. All this means that we can expect previously reported small effects of stressors and destressors (ie, factors reducing stress) on stress levels when working from home to be enhanced. Indeed, such results are currently starting to emerge in published literature, with findings that sometimes indicate increased stress responses [10-12] and sometimes reduced ones [13]; similarly, productivity is sometimes reduced [13,14] and sometimes increased [10]. This suggests a greater impact of stressors and destressors on stress levels when working from home during the pandemic.

When stress is prolonged, it can lead to mental and physical health issues, such as depression and burnout [15]. Burnout and even short-term stress lead to a decrease in overall performance and increased amounts of sick leave, costing companies billions of dollars every year [16]. The new hybrid working environment asks for accessible and dedicated solutions to help employees cope with their experienced stress at home. An effective solution would not only increase the mental well-being of employees but also significantly reduce costs for their employers. To create such solutions for stress management, insights into stressors or destressors when working from home are needed. A broad range of potential stressors and destressors have been included in recent research, most notably gender [10,12,17], age [17], the work-from-home experience [14,17], the presence of children [17], the fear of COVID-19 [11,17], work-life balance conflicts [17], social isolation [12,17], a distractive work environment [14,17], job characteristics [13], and sleep quality [12].

However, yet, by far, the greatest part of research on the relationship between stress and working from home has been panel studies with retrospective questionnaires [10-14,17]. No study has used unobtrusive measuring of stress levels, or in-the-moment polling of stress and other emotions through ecological momentary assessments (EMAs), a method of delivering short, recurring questionnaires at multiple times each day to tap into participant experiences without overburdening (see Ref. [18], page 98, for an overview of EMA use in a broad range of populations and research questions). In stress management, it is key to create solutions that require minimum effort for the employee to ensure extensive and effective use. The use of digital technologies that can sense and assess stress and its risk factors continuously, and EMAs for experience sampling, can be beneficial for both the growth of knowledge and the development of solution spaces.

The main aim of this study, therefore, was to find stressors and destressors when working from home using digital technologies (ie, questionnaires on a smartphone and physiological and activity state from a smartwatch). Both types of devices are increasingly prevalent in society and used to assess health. As a secondary objective, this study evaluated whether stressors or derivatives thereof can be assessed continuously and nonobtrusively by means of a smartwatch only. Continuous monitoring of stress and its risk factors on these devices would also allow for future intervention recommendations to avoid stressful moments, which could reduce the risk of developing long-term stress-related conditions [19].

The working-from-home conditions during the pandemic and the advancement and availability of smartwatches that measure physiology have created the possibility to conduct this study fully remote. Data of 202 participants from 3 different regions around the world (the United States, the United Kingdom, and Hong Kong) were collected within 12 weeks, without any physical meetings with the researchers and from the comfort of the participants’ own home.

## Methods

### Study Design

#### Recruitment

Participants were included if they were active employees of Cigna International (a global insurance company) at the time of the study and were working from home for at least 2 days a week. Only participants who met all inclusion criteria and none of the exclusion criteria (which can be found in Multimedia Appendix 1) could take part in the study. In total, 202 participants were included in the study: 50 (24.8%) in the United States, 70 (34.7%) in Hong Kong, and 82 (40.5%) in the United Kingdom. Participants were allowed to keep the Garmin smartwatch that they used in the study regardless of whether they had finished the study or stopped their participation before the end.

#### Procedure

**Figure 1:**
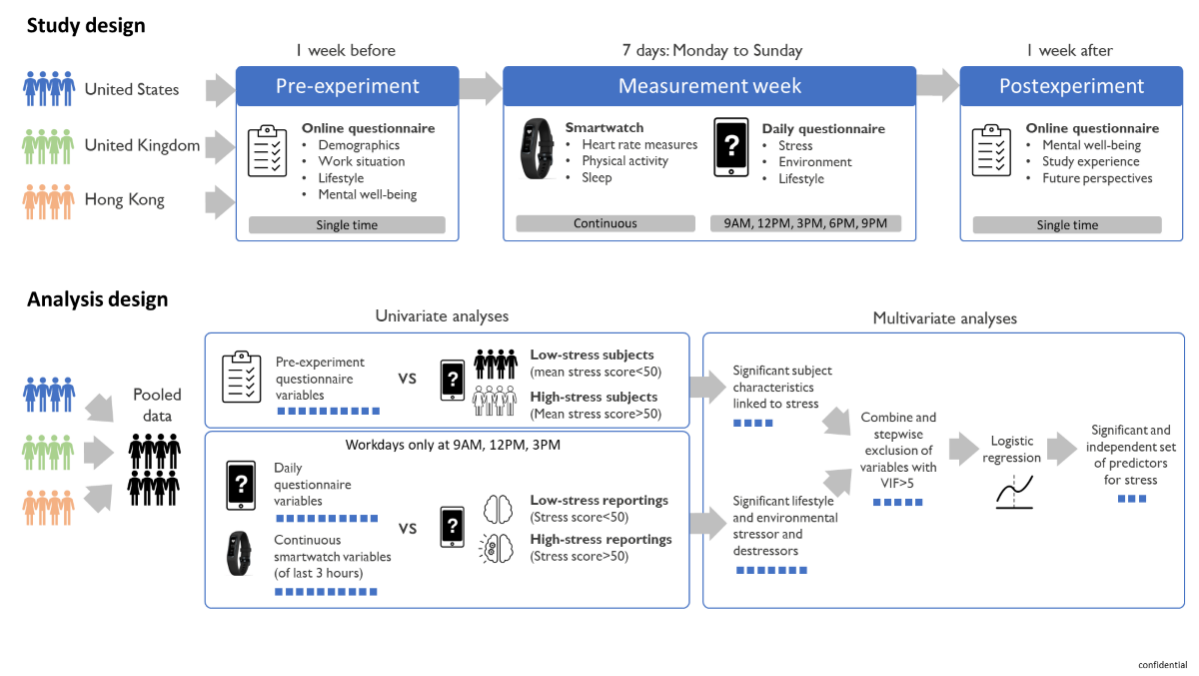
Study design (top) and analysis design (bottom). Blue squares in the analysis design represent a fictional number of variables remaining in each step. VIF: variance inflation factor.

The experiment was conducted in 12 weeks between April and September 2021. Measurements for the US participants were taken between April and June, UK participants between May and July, and Hong Kong participants in August. During these periods, there were no major COVID-19 infection peaks in any of the study regions; we therefore expected similar anxiety levels toward the virus during the entire measurement period [20]. Due to the COVID-19 pandemic, employees in all 3 regions were asked by their employers to work from home for at least a few days per week if work activities permitted this. The measurements lasted 7 days per participant, hereafter called “measurement week,” starting on Monday morning and ending on Sunday evening. A pre-experiment questionnaire was filled in the week before the measurement week, and a postexperiment questionnaire was filled in the week after the measurement week. See Figure 1 for a schematic overview of the study design.

During the measurement week, participants had to fill in the 5 daily questionnaires (EMAs) at set times. These EMAs were kept as short as possible to keep the participant burden as low as possible (see Ref. [21] for criteria). The EMAs were prompted in a questionnaire app on their smartphone. Participants wore the smartwatch continuously during the week (day and night) and got notifications in the questionnaire app to remind them to synchronize their watch to the smartwatch app on their phone.

#### Consumer Smartwatch

The Garmin Vivosmart 4 was used, which measures the user’s heart rate (HR), steps, classification of activity, sleep duration, sleep stages, and stress measurements, as derived by Garmin’s algorithms. Except for sleep characteristics [22,23] and stress measurements, the Garmin sensors have proven to be fairly accurate [24-27]. Participants had to download the Garmin Connect app to their own smartphone to set up and synchronize the data of the smartwatch during the study. The used smartwatch has a thin band and was therefore expected to not have any influence on the daily life of a participant.

#### Questionnaires

There were 3 sets of questionnaires: a pre-experiment questionnaire, a postexperiment questionnaire, and daily questionnaires (all questionnaires are available as Multimedia Appendix 2). The pre-experiment questionnaire contained questions on stable determinants and traits known from the literature to affect work-from-home stress: age; gender; job type; children present; work-from-home facilities (ie, a dedicated office), work-family interference, and social support from the boss, colleagues, and family members, respectively; current mental well-being; impact of the pandemic; and fear of (the debilitating consequences of) COVID-19. Moreover, the questionnaire contained questions on stress-mitigating activities, such as mindfulness and yoga, which are known to reduce work-related stress, both before [28,29] and during [17,30] the pandemic. The postexperiment questionnaire focused on participants’ experiences with the protocol and wearable device during the measurement week, their mental state of the past week, outlook on working from home, and stress detection by means of a wearable. The pre- and postexperiment questionnaires were filled in through Castor electronic data capture (EDC), a digital clinical trial platform to which participants got a link through their email.

Daily questionnaires were delivered 5 times a day using EMAs. To keep the participant burden acceptable, the questionnaires contained single questions on participants’ mental state (perceived stress, focus, motivation, productivity), the occurrence of stressors (work, children, house chores and other daily life distractions, noise, distractions from family, technical issues, others), and the occurrence of destressors (breaks, going outside, access to sunlight and fresh air, physical activity, social contacts). Morning questionnaires (9:00 a.m.) also contained questions on sleep quality and outlook on the upcoming day, whereas the midday questionnaires (noon, 3:00 p.m., and 6:00 p.m.) contained questions on current stress levels and activities and the mental state over the past 3 hours. The evening questionnaire (9:00 p.m.) had additional questions regarding the overall outlook on the past day, work times, and activities. Where possible, items were delivered as visual analog scales (VASs), a “gliding” scale ranging from “not at all” to “extremely,” which participants could seamlessly set to their preferences. VASs are at least as discriminating as questionnaires when it comes to highlighting differences in experience (see Ref. [31]), and known strengths of VASs are their relatively low burden for participants [32], their responsiveness to change [33], and their reliability when used repeatedly [34]. All daily questionnaires were delivered through the imec Q app, which also notified the user when a new questionnaire was ready. This questionnaire was only available for 90 minutes after it was prompted. The imec Q app was downloaded from the app store (iOS or Android) on the participants’ own smartphone, and registration to the study was done by a participant-specific quick response (QR) code.

### Ethical Considerations

This observational study was approved by the Western Institutional Review Board, USA (study number: 1303528; IRB tracking number: 20210874), and the Survey and Behavioral Research Ethics Committee at the Chinese University of Hong Kong, Hong Kong (study number: SBRE-20-798). No further approval was necessary for the United Kingdom. This study complies with the guidelines of the Declaration of Helsinki and is registered (registration ID NL9378) in the Dutch Trial Register (NTR).

To ensure anonymous participation of the subjects, recruitment emails were sent through the intranet of the company, asking employees to sign up by emailing the researchers from a personal email account of their choice. To make sure they understood the study design, participants had to correctly answer questions about the procedure of the study before being officially included in the study. All participants signed the informed consent form before participating in the study.

### Statistical Analysis

#### Sample Size

The sample size calculation was based on the aim of finding at least 1 known stressor. Research shows that 82.5% of people with high stress levels experience high noise in their environment [35]. Only 55.3% of people with low stress levels had high noise in their environment. Additionally, we used an outcome of a public opinion poll of 2019 where 38% of employees were estimated to experience stress in the workplace [36]. A 2-sample, 2-sided equality sample size calculation (p_A_=0.553, p_B_=0.825, k=0.62/0.38=1.632, 1 – β=.95, α=.05) resulted in a minimal sample size of 162. A power of 95% was chosen to account for the potential differences in the participant group per location. Accounting for dropout (10%) and loss of compliance during the week (10%), similarly to Smets et al [37], the actual required sample size of 194 was obtained.

#### Data

The data from the 3 different regions were combined to obtain the necessary sample size. Due to limited data of people working in the office and low compliance during the evenings and weekends, only daily data when working from home during work hours were used for analysis, including the prequestionnaire data. All data were divided into 3-hour blocks: 9:00 a.m.-noon, noon-3:00 p.m., and 3:00 p.m.-6:00 p.m. Each 3-hour block was associated with smartwatch data collected during the corresponding period and a questionnaire filled at the end of the period (at noon, 3:00 p.m., and 6:00 p.m.). Smartwatch data were averaged over the 3-hour period. Sleep quality from the 9:00 a.m. questionnaire was added to all other data points for that workday.

In the analysis, the stress level, as indicated by the participant, in the daily questionnaires on a VAS of 0 (no stress) to 100 (extremely stressed) was taken as the dependent variable, while the other daily questionnaire answers and smartwatch data were taken as independent variables. The independent variables were further divided into participant characteristics (ie, general demographics and environment) and environmental stressors (access to sunlight, access to fresh air, noise, distractions of daily life, and distractions of people), which were reported on a VAS of 0 (none) to 100 (a lot), and lifestyle stressors (taking a break, social interactions, going outside, and exercising), which were reported as a “yes” or “no” answer. Sleep quality was also part of the lifestyle stressors but was reported on a 0 (bad) to 100 (good) VAS. The smartwatch measurements of interest in the study were the HR, total sleep time, and activity data (step count and activity intensity). Activity intensity was scored by Garmin as “sedentary,” “active,” or “highly active” periods of maximally 15 minutes. No sleep stages were analyzed, due to the low or unknown performance in previous research characteristics [22,23]. Measurements from a 3-hour block were excluded in the analysis if either the smartwatch or the questionnaire data were missing. The percentage of excluded blocks is reported in the Results section.

#### Analysis and Modeling

In data exploration, a bimodal distribution of stress scores was observed (Multimedia Appendix 3). Furthermore, we found a relatively high frequency for stress scores of 50. Since the VAS was set to 50 by default in the questionnaires, there was a possibility of an unintentional increase of the scores corresponding to 50 in the case there was no answer from the participants or if the participants rushed though the questionnaire without moving the slider. The score distribution of various VAS variables also showed a relatively high frequency for the 50 values. We therefore excluded the exact value of 50 from the analysis, both for the stress scores and for the independent variables from the daily questionnaires. This exclusion emphasized the bimodal stress score distribution. Based on these findings and the increased interpretability of a binary outcome, we divided self-reported stress into 2 categories: low stress (stress scores lower than 50) and high stress (stress scores higher than 50). Furthermore, others have also reported robust findings after dichotomization of continuous questionnaire-based outcome variables [38,39] or binary stress classifications in wearable studies [40].

The variables from the pre-experiment questionnaire, daily questionnaire, and smartwatch were compared between the low- and high-stress groups by means of a univariate analysis and a multivariate analysis, as depicted in Figure 1.

Given the different data types for the low- and high-stress comparisons, different statistical tests were performed: a Mann-Whitney U (MWU) test for continuous/ordinal data, the Fisher exact test for 2 proportions, or a test of proportions for more than 2 proportions. The threshold for significance in all tests was set to *P*<.05.

Significant variables from the univariate analysis were used as input for the multivariate logistic regression. To minimize the effects of multicollinearity among the variables identified as participant characteristics, stressors, destressors, and smartwatch measurements in the univariate analysis, the variance inflation factor (VIF) was calculated. Stepwise reduction of variables was performed based on the VIF calculated on the initial set of significant variables; variables were dropped 1 by 1 (starting with the highest VIF) until a variable set with all VIFs less than 5 was obtained. A logistic regression model was trained on the resulting variables from the VIF analysis to classify instances as low stress (0) or high stress (1). The variables were scaled between 0 and 1 to make the coefficients comparable. Using the *P* values and coefficients obtained from the logistic regression model, the most important characteristics, stressors, destressors, and smartwatch measurements were identified (*P*<.05). All data analyses were performed using Python (version 3.8), Scikit-learn (version 1.0), and Scipy (version 1.8).

## Results

### Data Set Description

This study included 202 participants from 3 regions. This number of participants was divided over the 3 regions relative to the number of employees at those locations; see Table 1 for the exact number of participants per location.

Table 1 shows the characteristics of the sampling population. This study had a low rate of dropouts during the study (n=4, 2%). Dropouts were mainly caused by irritations from the watch or no specified reason. The low data availability in Hong Kong was caused by a bug in the Garmin data retrieval, which caused a loss of smartwatch data for 9 (13%) of 70 participants. Overall, population characteristics were similar across regions. Trends in differences of more female participants in United States and more anxiety toward the pandemic and more stress in Hong Kong were found.

**Table 1:** Population and data set characteristics, also split per region.

Characteristics			US participants (n=50)		UK participants (n=82)		Hong Kong participants (n=70)	Total participants (N=202)
Participants who completed the experiment, n (%)			47 (94)		82 (100)		69 (99)	198 (98)
Questionnaires completed during the workweek per participant (%), mean (SD)			92 (25)		80 (23)		73 (28)	81 (25)
Age (years), mean (SD)			43 (11.1)		41 (8.5)		38 (8.7)	40 (9.4)
**Gender, n (%)**								
	Male	12 (23)		36 (44)		30 (43)		79 (39)
	Female	38 (77)		46 (56)		40 (57)		123 (61)
**Role, n (%)^a^**								
	Manager	23 (46)		33 (40)		32 (46)		89 (44)
	Analyst	12 (23)		25 (31)		15 (21)		51 (25)
	Customer service	5 (10)		9 (11)		1 (1)		16 (8)
	Other	11 (21)		15 (18)		22 (32)		46 (23)
Participants with children aged <10 years in the house during the workday, n (%)			11 (21)		25 (30)		16 (23)	51 (25)
Job very to extremely demanding/intense, n (%)			17 (33)		36 (44)		27 (38)	81 (40)
Moderate-to-extreme anxiety toward COVID-19, n (%)			18 (35)		23 (28)		36 (52)	79 (39)
Stress level throughout the workweek (0-100), mean (SD)			34.5 (26.2)		36.2 (27.7)		43.8 (22.8)	37.5 (26.4)

^a^The numbers could be more than the total because of rounding.

### Participant Characteristics Related to Stress

Table 2 shows the statistics to indicate an enrichment of pre-experiment questionnaire variables for either the low- or the high-stress group based on the entire sampling population. Burnout experience, job intensity, whether family and everyday life events get in the way of work, and anxiety due to the pandemic were all significantly different between low- and high-stress groups. There was no significant difference in region between low and high stress levels.

**Table 2:** Differences in stress levels for participant profile items based on the entire population^a^.

Profile items		Low stress	High stress	Ratio	*P* value	Test
Age (years)		Mean 40.9 (SD 9.0)	Mean 38.4 (SD 9.4)	0.94	.10	*U* ^b^
Gender, male/female		0.39	0.31	0.81	.39	*F* ^c^
Children, yes/no		0.35	0.26	0.75	.35	*F*
Dedicated office space, yes/no		0.81	0.74	0.90	.34	*F*
Experienced burnout in the past, yes/no		0.45	0.76	1.69	.03^d^	*F*
Perform stress reduction activities, yes/no		0.48	0.64	1.32	.09	*F*
Family/life events get in the way of work (0-4)		Mean 1.1 (SD 0.9)	Mean 1.3 (SD 0.8)	1.25	.05^d^	*U*
Support from colleagues (0-4)		Mean 2.7 (SD 1.0)	Mean 2.4 (SD 1.0)	0.91	.08	*U*
Job intensity (0-4), mean (SD)		Mean 2.2 (SD 0.8)	Mean 2.6 (SD 0.8)	1.18	.01^d^	*U*
Relationship with colleagues (0-4)		Mean 3.0 (SD 0.7)	Mean 2.8 (SD 0.5)	0.94	.08	*U*
Life affected by pandemic (0-4)		Mean 2.2 (SD 0.9)	Mean 2.4 (SD 0.7)	1.08	.17	*U*
Living a full life during the pandemic (0-4)		Mean 1.6 (SD 1.0)	Mean 1.7 (SD 1.0)	1.04	.67	*U*
Help from others in dealing with life (0-4)		Mean 1.9 (SD 1.1)	Mean 1.9 (SD 1.2)	0.98	.95	*U*
Anxiety due to pandemic (0-4)		Mean 1.2 (SD 0.8)	Mean 1.4 (SD 0.9)	1.24	.04^d^	*U*
**Region**						
	Hong Kong (n=59)	38 (64%)	21 (36%)	0.55	.10	Pr^e^
	United Kingdom (n=82)	65 (79%)	17 (21%)	0.26	.10	Pr
	United States (n=43)	34 (79%)	9 (21%)	0.26	.10	Pr

^a^The “Low stress” and “High stress” columns represent the mean (SD) values for continuous/ordinal variables, such as age, and a ratio of the participant count for binary variables, such as gender. The “Ratio” column represents the ratio of these values between high and low stress levels. Responses ranged from 0 for very low/little to 4 for very high/much. The *P* value is for the performed test that is depicted in the “Test” column.

^b^*U*: Mann-Whitney *U* (MWU) test.

^c^*F*: Fisher exact test.

^d^*P*<.05

^e^Pr: test of proportions.

### Identification of Stressors and Destressors

**Figure 2:**
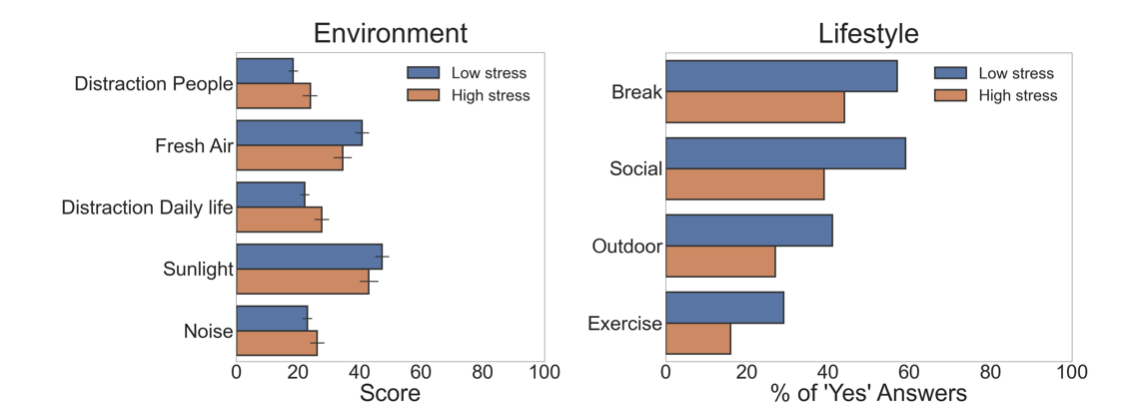
Significant environmental and lifestyle stressors and destressors subjectively scored in the daily questionnaires (*P*<.05). The bars represent the mean value of the group, and the error bars on the environmental stressors represent confidence.

Environmental and lifestyle factors that were subjectively scored 3 times per workday were compared between high- and low-stress groups. The results of these comparisons are depicted in Table 3. Significant stressors and destressors are also visualized in Figure 2. The significant environmental stressors when working from home were distractions of other people, distractions of daily life, and noise of the surroundings. The significant environmental lifestyle destressors were access to fresh air and sunlight, and the lifestyle destressors were taking a break, having social interactions outside of work, going outside, and exercising. There was no significant relationship found between low and high stress levels during work hours and the quality of sleep during the night before.

**Table 3:** Ratio of values of variables between low- and high-stress groups and the coefficients of stress prediction modeling^a^.

Characteristics		Univariate analysis					Multivariate analysis			
		Low stress	High stress	Ratio	*P* value	Test	VIF^b^ initial	VIF final	B (LR)^c^	*P* value
**Participant characteristics**										
	Family/life events get in the way of work (0-4)	Mean 1.1 (SD 0.9)	Mean 1.3 (SD 0.8)	1.25	.05^d^	*U* ^e^	3.46	2.24	1.18	<.001^f^
	Job intensity (0-4)	Mean 2.2 (SD 0.8)	Mean 2.6 (SD 0.8)	1.18	.007^d^	*U*	9.97	N/A^g^	N/A	N/A
	Anxiety due to pandemic (0-4)	Mean 1.2 (SD 0.8)	Mean 1.4 (SD 0.9)	1.24	.04^d^	*U*	7.69	N/A	N/A	N/A
	Experienced burnout in the past, yes/no	0.45	0.76	1.69	.03^d^	*F* ^h^	4.98	3.37	1.08	<.001^f^
**Environment**										
	Sunlight (0-100)	Mean 47.2 (SD 32.3)	Mean 42.9 (SD 30.4)	0.91	.02^d^	*U*	5.18	4.99	0.18	.49
	Fresh air (0-100)	Mean 40.7 (SD 33.6)	Mean 34.5 (SD 31.7)	0.84	<.001^f^	*U*	4.99	4.93	–0.08	.78
	Noise (0-100)	Mean 23.0 (SD 23.9)	Mean 26.2 (SD 25.0)	1.13	.008^d^	*U*	2.70	2.65	0.08	.79
	Distraction of daily life (0-100)	Mean 22.2 (SD 23.4)	Mean 27.7 (SD 26.7)	1.24	<.001^f^	*U*	3.50	3.49	0.23	.51
	Distraction of people (0-100)	Mean 18.4 (SD 22.2)	Mean 23.9 (SD 26.0)	1.30	<.001^f^	*U*	3.22	3.21	0.52	.17
**Lifestyle**										
	Social, yes/no	0.59	0.39	0.66	<.001^f^	*F*	2. 27	2.23	–0.89	<.001^f^
	Outdoor, yes/no	0.41	0.27	0.66	<.001^f^	*F*	2.86	2.85	–0.44	.03^d^
	Break, yes/no	0.57	0.44	0.77	<.001^f^	*F*	2.47	2.40	–0.03	.83
	Exercise, yes/no	0.29	0.16	0.55	<.001^f^	*F*	2.37	2.35	–0.28	.20
	Sleep quality of night before (0-100)	Mean 61.9 (SD 27.7)	Mean 61.5 (SD 27.1)	0.99	.64	*U*	N/A	N/A	N/A	N/A
**Smartwatch**										
	Active intensity (0-12)	Mean 7.92 (SD 3.0)	Mean 5.99 (SD 2.9)	0.85	<.001^f^	*U*	5.28	4.57	0.18	.83
	Highly active intensity (0-12)	Mean 1.87 (SD 2.2)	Mean 1.29 (SD 1.8)	0.83	.04^d^	*U*	1.84	1.68	0.37	.37
	Steps (count)	Mean 1858 (SD 1863)	Mean 875 (SD 1203)	0.66	<.001^f^	*U*	4.65	4.07	–0.27	.62
	Mean HR^i,j^ (beats per minute [bpm])	Mean 78.78 (SD 9.5)	Mean 78.35 (SD 9.4)	0.99	.57	*U*	N/A	N/A	N/A	N/A
	SD HR^k^ (bpm)	Mean 10.9 (SD 6.5)	Mean 9.0 (SD 3.9)	0.82	<.001^f^	*U*	9.01	N/A	N/A	N/A
	Sleep duration (hours)	Mean 7.7 (SD 1.0)	Mean 7.8 (SD 1.2)	1.01	.66	*U*	N/A	N/A	N/A	N/A

^a^The “Low stress“ and “High stress” columns represent the mean (SD) values. The “Ratio” column represents the ratio between high and low stress levels. Yes/no questions are presented as the ratio of “yes” answers. Responses ranged from 0 for very low/little to 4 for very high/much.

^b^VIF: variance inflation factor.

^c^B(LR): binary (logistic regression) weight.

^d^*P*<.05.

^e^*U*: Mann-Whitney *U* (MWU) test.

^f^*P*<.001.

^g^N/A: not applicable.

^h^*F*: Fisher exact test.

^i^HR: heart rate.

^j^The “Low stress” and “High stress” values represent the mean and SD of the mean HR feature within the specified time window.

^k^The “Low stress” and “High stress” values represent the mean and SD of the SD HR feature within the specified time window.

### Measurements With the Smartwatch

**Figure 3:**
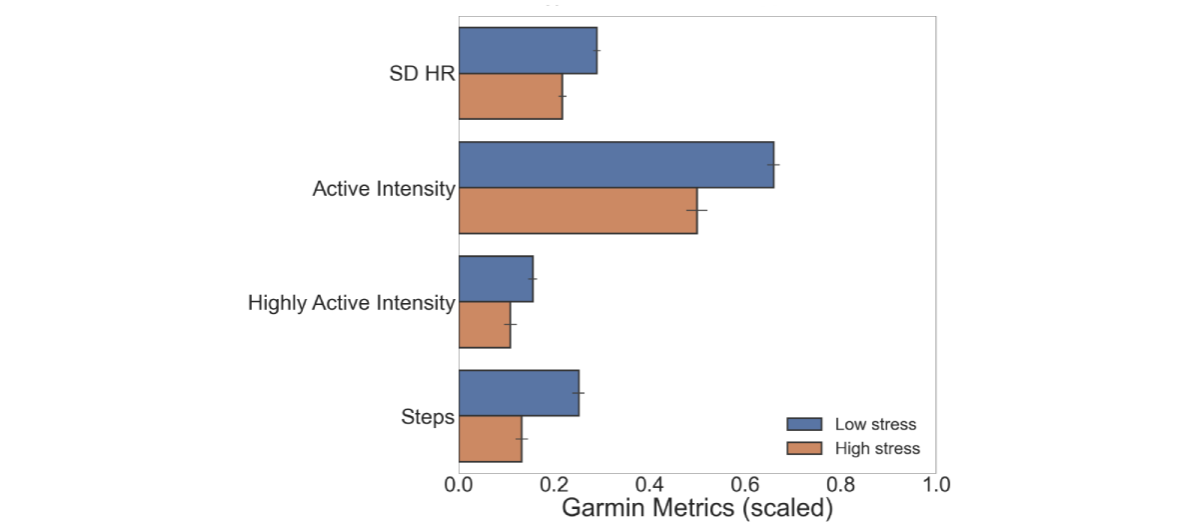
Significant activity and physiological predictors of stress from the smartwatch with *P*<.05. The bars represent the mean value of the stress group, and the error bars represent CIs of the mean. The metrics are scaled to values between 0 and 1 for visualization purposes. HR: heart rate.

The smartwatch measured and calculated variables for physical activity, HR, and sleep duration. We excluded 14% of 3-hour data blocks of questionnaire data due to missing smartwatch data. Table 3 shows the results of the comparisons made between low- and high-stress groups, and significant predictors are visualized in Figure 3. The SD of the HR, active and highly active intensity periods, and the number of steps of the past 3 hours were associated with stress. No significant relationships were found between the stress levels and the average HR and sleep duration.

### Combining Predictors for Stress

All identified variables from participant characteristics, lifestyle, environment, and smartwatch that were significantly associated with stress were combined in a multivariate analysis to identify a set of independent predictors of stress. By calculating the VIF over this set of variables, 5 (29%) of 17 predictors (with VIF>5) were identified as predictors contributing significantly to multicollinearity (Table 3). Stepwise reduction of variables with VIF>5 resulted in a set of 14 variables that was used as input for binary logistic regression. Of these input variables, 4 (29%) were found significantly associated with stress in the regression model and they were all self-reported in the pre-experiment questionnaire or daily lifestyle questions. Higher stress was significantly associated with more disruptive family or life events while working (*P*<.001), previous experience with burnout (*P*<.001), fewer social events (*P*<.001), and less outdoor time (*P*=.03).

Since all multivariate predictors for stress result from questionnaire data, the question arises whether continuous and nonobtrusive sensing of environmental conditions and human activity/physiology can replace repetitive questionnaires. To answer that question, we ran 2 logistic regression models with a 3-fold cross-validation scheme: one reference model with the significant questionnaire predictors from the original logistic regression (whether they had disruptive family or life events while working, experience with burnout, social events, and outdoor events) and another model with predictors that can be (potentially) continuously and passively sensed (fresh air, noise, sunlight, steps, SD of the HR, active intensity, highly active intensity, and the 2 participant characteristics of whether they had disruptive family or life events and experience with burnout). The questionnaire model resulted in *F*_1_ scores of 0.66, 0.4, and 0.48 in the 3-fold cross-validation (mean *F*_1_=0.51, SD *F*_1_=0.11). The continuous sensing model resulted in *F*_1_ scores of 0.59, 0.38, and 0.44, which were not much lower than those for the questionnaire model (mean *F*_1_=0.47, SD *F*_1_=0.09). In addition, both models were better than guessing randomly using known class distributions (mean *F*_1_=0.33) or by majority class guessing (mean *F*_1_=0). This indicates that continuous and nonobtrusive sensing has the potential to replace questionnaires when monitoring momentary stress.

### Bidirectional Relationship Between Stress and Sleep

Results in Table 3 show that the sleep quality of the night before, as answered in the 9:00 a.m. daily questionnaires, was not associated with stress levels across an entire workday, the same counts for the smartwatch measurements of sleep duration. We additionally checked whether sleep quality affected experienced stress levels reported in the 9:00 a.m. questionnaire on all measured days (workdays and non-workdays). The average sleep quality was significantly higher (*P*<.001, MWU test) in the low stress samples (Mean: 66.8, *SD*: 26.3) than in the high stress samples (Mean: 55.7, *SD*: 28.5). Similarly, we checked whether stress reported at the end of the day (9:00 P.M.) was associated with sleep quality in the following night across all measured days. Low stress moments resulted in significantly (*P*=.004, MWU test) higher sleep quality scores (mean 65.1, SD 26.6) compared to the high-stress moments (mean 59.7, SD 26.8). These results indicate that high evening stress is related to poor sleep quality during the night after and that low sleep quality is correlated with higher-stress moments in the morning after.

## Discussion

### Principal Findings

This study had 2 main objectives: first, using EMAs to sample the effect of stressors on work-from-home–related mental well-being. The study showed that participants working from home had stressors and destressors related to their characteristics, environment, and lifestyle. Family and daily life getting in the way of work, job intensity, a history of burnout, and anxiety toward the COVID-19 pandemic were all related to higher stress levels. Environmental stressors were noise and distractions from both daily lives as well as other people. The destressors found to influence the stress levels of the participants were having access to sunlight and fresh air during workhours. Further destressors related to lifestyle were going outdoors, taking breaks, exercising, and having social interactions outside of work. These stressors and destressors are comparable to those found in previous research [17,41], reporting that decreased physical activity, social isolation, family-work conflict, and more distractions when working from home are significant predictors of decreased mental well-being. However, not all potential determinants in previous research showed to affect mental well-being were significant predictors in this study. Most notably, age, gender, the presence of children, a dedicated office space, social support from colleagues, impact of the pandemic, and sleep quality did not significantly impact stress levels. Further research, especially meta-analyses, should shed light upon the causes and value of these results. A major difference between this study and the greater part of previous research is that the latter made use of single, retrospective questionnaires, whereas this study applied recurrent, brief measurements to sample current experiences. Further research using experience sampling methodology, such as EMAs, could further elucidate the impact of measurement timing on determinant strength.

The second objective was to compare EMA measurements to more unobtrusive physiological measurements with a smartwatch. Our results indeed suggest that some of the activity-related stressors and destressors found in this study could be indirectly measured with a smartwatch. The significant activity measurements of the smartwatch, such as steps and activity classifications, were negatively related to stress and may represent destressors, such as exercise, breaks taken, and going outside, as these could all be represented by movement. The same reasoning can be applied for the significant negative relationship between the SD of the HR and stress. A potential hypothesis is that the HR will fluctuate more during activity compared to sitting still behind a desk, which is associated with lower stress levels, with the important notion that these associations were not tested for causality.

When all significant participant characteristics, stressors, destressors, and smartwatch measurements were combined into a model, only some participant characteristics and destressors remained significant in stress prediction. Two participant characteristics significantly contributed to the stress prediction model, namely the disruption of family and everyday life events and the previous experience of burnout. The former could be a direct effect of the working-from-home regime, where families are asked to stay and work from home to limit the spread of the COVID-19 virus, causing personal and work lives to no longer be separate. The latter could be a residual effect of low stress tolerance during the recovery of stress-related exhaustion [42]. These residual effects are shown to still be prevalent after at least 7 years since seeking help. Future research should examine whether these participant characteristics persist being significant or whether their influence is reduced due to the opening of schools, offices, and other locations. Two destressors significantly contributed to the model: having social interactions and being outdoors. These findings may indicate that encouraging employees to go outside during breaks and talking to people outside of work meetings could already have a beneficial effect on their mental state.

### Wearables and Stress

This study showed a minimal influence of smartwatch data in the most optimal model when added to the questionnaire data. This redundancy could be the result of the questionnaires already covering the amount of time spent outdoors, for example, which would also be represented in the steps, active periods, and highly active periods measured by the smartwatch. Although performances for the prediction model based on questionnaire data alone and the model that uses smartwatch and contextual data were relatively low (*F*_1_ score of 0.66 and 0.59, respectively), their similar performance could indicate that in the future, in potential, questionnaire measurements can be replaced by wearable or other unobtrusive sensor measurements.

Ideally, someone’s stressors and destressors could be automatically measured with a smartwatch such that no effort is needed from the employee to gather data of their mental state and surroundings. Even though the current state of smartwatches might not be able to inform accurate stress prediction models by themselves, it is expected that performance will improve in the future. As companies are adding better and more sensors to their smartwatches in nearly every new version, it is likely that more stressors and destressors can be measured automatically. Additional physiological sensors, such as skin conductance, skin temperature, and HR variability, could improve stress detection [37], while additional environmental sensors, such as sound levels and ambient light sensors, could measure noise, distractions, the environment, and outdoor activities and therefore replace questions to the user. Furthermore, additional sensing technology, such as smart chairs [43], smart glasses, office equipment use [44], and data collected with a smartphone [45] could improve stress detection.

However, future studies concerning the accuracy of devices in measuring physiological signals need to be conducted as previous studies have shown a wide variability in the accuracy of different consumer devices [22,46-48]. Furthermore, more data must be collected to build reliable models for stress detection. So far, stress prediction models by means of wearables have shown promising, although not 100% accurate, results in a controlled environment with momentary stress tasks [49]. However, stress levels in daily life can be different from those in a controlled laboratory setting. Consequently, model performance of studies in daily life settings is lower [37,40,50]. Next steps in this field of research would need to focus on detecting stress by means of more and improved unobtrusive measurements in a daily dynamic setting. If stress detection models would eventually be reliable enough, it would provide the possibility to monitor stress levels and intervene when high stress levels are detected in order to prevent stress-related diseases in the long term.

### Limitations

This study consisted of 202 participants from 3 different regions of the world: the United States, the United Kingdom, and Hong Kong. As Table 1 has shown, there are some differences between Hong Kong, the United States, and the United Kingdom, specifically in average stress levels reported and anxiety toward the COVID-19 pandemic in Hong Kong participants. Although these 2 can be related to each other, the reason could also be cultural differences. Cultural differences in norms and values might cause a difference in the definition or the view of mental health [51], which makes reported mental health scores hard to compare between regions. The higher stress levels in Hong Kong might also be due to the specific company location, which can be caused by a difference in organizational structures or the intensity of the assignments to be performed. The same counts for the anxiety toward the pandemic, as COVID-19 measures differed per region. Even though location (with their cultural and societal differences) might affect stress levels, we were still able to find common stressors and destressors, possibly due to the robust split in low- and high-stress groups. Regional differences in stress scores also did not differ significantly in any of the statistical tests. Furthermore, a difference between regions was accounted for in the sample size calculation by using a high power of 0.95.

Another limitation of this study is that the participant group consisted of employees of the same company, as certain stressors and destressors might be specific to this company. However, we expect the influence to be minimal. These employees were chosen due to the international character of Cigna International, representing multiple cultural backgrounds, and the wide variety of roles within the company. In addition, most employees went from working full-time in the office to working from home overnight similarly to a large part of the population. In addition, similar research among other companies has shown similar stressors and destressors when working from home [17,41], showing similar influences on stress in different working environments. Furthermore, the significant stressors and destressors found in this study are largely unrelated to the job itself, except job intensity. However, job intensity has been proven to cause stress in a variety of job sectors worldwide [52-54]. Therefore, we expect similar results to be obtained performing this study with employees of different companies.

For many of the single-item measures used in the questionnaires, a broad range of longer, validated versions exist [55], such as the Perceived Stress Scale (PSS) [56]. However, we opted not to use those in order to reduce participant burden; especially in recurrent questionnaires, longer answering times and higher burden are known to significantly reduce adherence [18,21]. This can be seen as a limitation of this study because unvalidated questionnaires could potentially be less valid and reliable. However, the literature shows that single-item questions, especially the VASs used in this study, are well suited to measure a multitude of constructs, often as effectively as longer, multi-item questionnaires [57,58]. We therefore argue that the current questionnaires, with shorter, repetitive measurements, often in the form of VASs, have sufficient reliability and validity to aptly sample participants’ experiences. Future replications and further studies can support (or, of course, reject) this assumption.

Finally, the dichotomous split in stress scores can be argued. Our first argument is that the stress score distributions were bimodal in nature. Second, a clear and interpretable outcome of both univariate and multivariate analyses was preferred, also to standardize the analysis design. These arguments were considered having more weight than a potential slight loss of statistical power that dichotomization may cause. Third, previous studies have followed a similar dichotomous approach to continuous questionnaire-based outcomes and have made robust findings [38,39].

### Remote Study

This study showed that valuable results can be obtained in research that is conducted fully remote in different regions of the world. Participants lived their normal daily lives and participated in the study from the comfort of their own homes, without any effort to go to a specific research location, therefore minimizing moderator and recall bias. They always had the possibility of asking questions and requesting videocalls, and data were monitored by the researchers to make sure everything was still working. Even though contact was limited, the dropout rate was low and the average percentage of filled-in questionnaires per participant was high (81%). Furthermore, many participants could be measured at the same time, significantly reducing time investments of the researchers and also reducing the influence of the measurement period. In this case, all participants had to be measured within the same COVID-19 time frame to have comparable situations for all participants. However, even future studies will benefit from simultaneous measurements. It is shown that mental states are affected by outside temperature or the duration of sunlight during the day [59,60], and measuring all participants within a short time frame could limit this effect.

These results show that the fast advancement of digital technology, including wearables or other unobtrusive sensors, secure communication platforms, and smartphone applications, extend our possibilities of conducting daily-life research with large sample sizes. A small note must be made in that relying on technologies also brings dependencies on the reliability of that technology as access to backups or support is often limited. This was demonstrated by the smartwatch issue we had in Hong Kong, causing us to lose a small part of data in that region.

### Conclusion

This study shows that people have different stressors and destressors when working from home; these should be considered when managing stress in employees. Some of these stressors and destressors are (in)directly measurable with unobtrusive sensors, and prediction models based on these data show promising results for the future of automatic stress detection and management.

These findings may contribute to people optimizing the home office environment (enough sunlight, fresh air, and low noise levels) and activity suggestions during work hours (having social interactions, going outside, and exercising during breaks) to reduce stress levels.

## Appendix

Multimedia Appendix 1 Inclusion and exclusion criteria.

Multimedia Appendix 2 Questionnaires: the general inclusion questionnaire, the daily questionnaire, and the pre- and postexperiment questionnaires.

Multimedia Appendix 3 Distributions of the answers to 6 of the daily questionnaires.

## Abbreviations

EMA: ecological momentary assessment
HR: heart rate
MWU: Mann-Whitney U
VAS: visual analog scale
VIF: variance inflation factor

## References

[1] World Health Organization (2021). Listings of WHO's Response to COVID-19.

[2] Salari N, Hosseinian-Far A, Jalali R, Vaisi-Raygani A, Rasoulpoor S, Mohammadi M, Rasoulpoor S, Khaledi-Paveh B (2020). Prevalence of stress, anxiety, depression among the general population during the COVID-19 pandemic: a systematic review and meta-analysis. Global Health.

[3] Meta Careers Meta.

[4] Google A Hybrid Approach to Work.

[5] PwC The Benefits and Challenges of Hybrid Working.

[6] Mann S, Holdsworth L (2003). The psychological impact of teleworking: stress, emotions and health. New Technol Work Employ.

[7] Song Y, Gao J (2019). Does telework stress employees out? A study on working at home and subjective well-being for wage/salary workers. J Happiness Stud.

[8] Russell HO (2009). The impact of flexible working arrangements on work-life conflict and work pressure in Ireland. Gender Work Organ.

[9] Sullivan C (2012). Remote working and work-life balance. Work and Quality of Life.

[10] Sandoval-Reyes J, Idrovo-Carlier S, Duque-Oliva EJ (2021). Remote work, work stress, and work-life during pandemic times: a Latin America situation. Int J Environ Res Public Health.

[11] Shao Y, Fang Y, Wang M, Chang C(, Wang L (2021). Making daily decisions to work from home or to work in the office: the impacts of daily work- and COVID-related stressors on next-day work location. J Appl Psychol.

[12] Şentürk E, Sağaltıcı E, Geniş B, Günday Toker Ö (2021). Predictors of depression, anxiety and stress among remote workers during the COVID-19 pandemic. WOR.

[13] Shimura A, Yokoi K, Ishibashi Y, Akatsuka Y, Inoue T (2021). Remote work decreases psychological and physical stress responses, but full-remote work increases presenteeism. Front Psychol.

[14] Kumar P, Kumar N, Aggarwal P, Yeap JA (2021). Working in lockdown: the relationship between COVID-19 induced work stressors, job performance, distress, and life satisfaction. Curr Psychol.

[15] Lindblom KM, Linton SJ, Fedeli C, Bryngelsson I (2006). Burnout in the working population: relations to psychosocial work factors. Int J Behav Med.

[16] Hassard J, Teoh KRH, Visockaite G, Dewe P, Cox T (2018). The cost of work-related stress to society: a systematic review. J Occup Health Psychol.

[17] Galanti T, Guidetti G, Mazzei E, Zappala S, Toscano F (2021). Work from home during the covid-19 outbreak: the impact on employees' remote work productivity, engagement, and stress. J Occup Environ Med.

[18] Myin-Germeys I, Kuppens P (2022). The Open Handbook of Experience Sampling Methodology: A Step-by-Step Guide to Designing, Conducting, and Analyzing ESM Studies. 2nd ed.

[19] Heber E, Lehr D, Ebert DD, Berking M, Riper H (2016). Web-based and mobile stress management intervention for employees: a randomized controlled trial. J Med Internet Res.

[20] World Health Organization WHO Coronavirus (COVID-19) Dashboard.

[21] Eisele G, Vachon H, Lafit G, Kuppens P, Houben M, Myin-Germeys I, Viechtbauer W (2022). The effects of sampling frequency and questionnaire length on perceived burden, compliance, and careless responding in experience sampling data in a student population. Assessment.

[22] Stone JD, Rentz LE, Forsey J, Ramadan J, Markwald RR, Finomore VS, Galster SM, Rezai A, Hagen JA (2020). Evaluations of commercial sleep technologies for objective monitoring during routine sleeping conditions. Nat Sci Sleep.

[23] Mouritzen NJ, Larsen LH, Lauritzen MH, Kjær TW (2020). Assessing the performance of a commercial multisensory sleep tracker. PLoS One.

[24] Claes J, Buys R, Avila A, Finlay D, Kennedy A, Guldenring D, Budts W, Cornelissen V (2017). Validity of heart rate measurements by the Garmin Forerunner 225 at different walking intensities. J Med Eng Technol.

[25] Lamont RM, Daniel HL, Payne CL, Brauer SG (2018). Accuracy of wearable physical activity trackers in people with Parkinson's disease. Gait Posture.

[26] Reddy RK, Pooni R, Zaharieva DP, Senf B, El Youssef J, Dassau E, Doyle Iii FJ, Clements MA, Rickels MR, Patton SR, Castle JR, Riddell MC, Jacobs PG (2018). Accuracy of wrist-worn activity monitors during common daily physical activities and types of structured exercise: evaluation study. JMIR Mhealth Uhealth.

[27] Lee J, Byun W, Keill A, Dinkel D, Seo Y (2018). Comparison of wearable trackers' ability to estimate sleep. Int J Environ Res Public Health.

[28] Murphy LR (1996). Stress management in work settings: a critical review of the health effects. Am J Health Promot.

[29] Janssen M, Heerkens Y, Kuijer W, van der Heijden B, Engels J (2018). Effects of mindfulness-based stress reduction on employees' mental health: a systematic review. PLoS One.

[30] Zadok-Gurman T, Jakobovich R, Dvash E, Zafrani K, Rolnik B, Ganz AB, Lev-Ari S (2021). Effect of inquiry-based stress reduction (IBSR) intervention on well-being, resilience and burnout of teachers during the covid-19 pandemic. Int J Environ Res Public Health.

[31] Lesage F, Berjot S, Deschamps F (2012). Clinical stress assessment using a visual analogue scale. Occup Med (Lond).

[32] McCormack HM, Horne DJ, Sheather S (1988). Clinical applications of visual analogue scales: a critical review. Psychol Med.

[33] Huskisson EC, Jones J, Scott PJ (1976). Application of visual-analogue scales to the measurement of functional capacity. Rheumatol Rehabil.

[34] Bowsher D, Wells P, Frampton V, Bowsher D (1994). Acute and chronic pain and assessment. Pain Management by Physiotherapy. 2nd ed.

[35] Mursali A, Endang B, Dharmono S (2009). Relationship between noise and job stressat a private thread spinning company. Universa Medicina.

[36] Gallup State of the Global Workplace: 2022 Report.

[37] Smets E, Rios Velazquez E, Schiavone G, Chakroun I, D'Hondt E, De Raedt W, Cornelis J, Janssens O, Van Hoecke S, Claes S, Van Diest I, Van Hoof C (2018). Large-scale wearable data reveal digital phenotypes for daily-life stress detection. NPJ Digit Med.

[38] Cho Y, Julier SJ, Bianchi-Berthouze N (2019). Instant stress: detection of perceived mental stress through smartphone photoplethysmography and thermal imaging. JMIR Ment Health.

[39] Van Der Feltz-Cornelis CM, Varley D, Allgar VL, de Beurs E (2020). Workplace stress, presenteeism, absenteeism, and resilience amongst university staff and students in the covid-19 lockdown. Front Psychiatry.

[40] Han HJ, Labbaf S, Borelli JL, Dutt N, Rahmani AM (2020). Objective stress monitoring based on wearable sensors in everyday settings. J Med Eng Technol.

[41] Xiao Y, Becerik-Gerber B, Lucas G, Roll SC (2021). Impacts of working from home during COVID-19 pandemic on physical and mental well-being of office workstation users. J Occup Environ Med.

[42] Glise K, Wiegner L, Jonsdottir IH (2020). Long-term follow-up of residual symptoms in patients treated for stress-related exhaustion. BMC Psychol.

[43] Hyun Jae Baek, Gih Sung Chung, Ko Keun Kim, Kwang Suk Park (2012). A smart health monitoring chair for nonintrusive measurement of biological signals. IEEE Trans Inform Technol Biomed.

[44] Pepa L, Sabatelli A, Ciabattoni L, Monteriu A, Lamberti F, Morra L (2021). Stress detection in computer users from keyboard and mouse dynamics. IEEE Trans Consumer Electron.

[45] Stutz T, Blechert J, Tiefengrabner M, Wilhelm F (2015). Smartphone based stress prediction.

[46] Guo F, Li Y, Kankanhalli M, Brown M (2013). An evaluation of wearable activity monitoring devices.

[47] Case MA, Burwick HA, Volpp KG, Patel MS (2015). Accuracy of smartphone applications and wearable devices for tracking physical activity data. JAMA.

[48] Gillinov S, Etiwy M, Wang R, Blackburn G, Phelan D, Gillinov AM, Houghtaling P, Javadikasgari H, Desai M (2017). Variable accuracy of wearable heart rate monitors during aerobic exercise. Med Sci Sports Exerc.

[49] Han L, Zhang Q, Chen X, Zhan Q, Yang T, Zhao Z (2017). Detecting work-related stress with a wearable device. Comput Ind.

[50] Muaremi A, Arnrich B, Tröster G (2013). Towards measuring stress with smartphones and wearable devices during workday and sleep. Bionanoscience.

[51] Oishi S (2018). Culture and subjective well-being: conceptual and measurement issues. Handbook of Well-Being.

[52] Spielberger C, Vagg P, Wasala C, Quick JC, Tetrick LE (2003). Occupational stress: job pressures and lack of support. Handbook of Occupational Health Psychology.

[53] Chiang FF, Birtch TA, Kwan HK (2010). The moderating roles of job control and work-life balance practices on employee stress in the hotel and catering industry. Int J Hosp Manag.

[54] Elovainio M, Heponiemi T, Kuusio H, Jokela M, Aalto A, Pekkarinen L, Noro A, Finne-Soveri H, Kivimäki M, Sinervo T (2015). Job demands and job strain as risk factors for employee wellbeing in elderly care: an instrumental-variables analysis. Eur J Public Health.

[55] Cooke PJ, Melchert TP, Connor K (2016). Measuring well-being: a review of instruments. Couns Psychol.

[56] Cohen S, Kamarck T, Mermelstein R (1983). A global measure of perceived stress. J Health Soc Behav.

[57] Huang Z, Kohler IV, Kämpfen F (2020). A single-item Visual Analogue Scale (VAS) measure for assessing depression among college students. Community Ment Health J.

[58] Bergkvist L, Rossiter JR (2018). The predictive validity of multiple-item versus single-item measures of the same constructs. J Mark Res.

[59] Liu J, Varghese BM, Hansen A, Xiang J, Zhang Y, Dear K, Gourley M, Driscoll T, Morgan G, Capon A, Bi P (2021). Is there an association between hot weather and poor mental health outcomes? A systematic review and meta-analysis. Environ Int.

[60] Holick M (2001). A perspective on the beneficial effects of moderate exposure to sunlight: bone health, cancer prevention, mental health and well-being. Compr Ser Photosci.

