# Supplementary material for: Stressors and Destressors in Working From Home Based on Context and Physiology From Self-Reports and Smartwatch Measurements: International Observational Study Trial
Source: JMIR Form Res. 2022 Nov 10;6(11):e38562. doi: 10.2196/38562 (PMC9651003; doi:10.2196/38562)
Supplement: Multimedia Appendix 1 [file formative_v6i11e38562_app1.docx]

# Multimedia Appendix 1

## Inclusion criteria

- age between 18 and 65 years old
- provide consent to participate
- work for at least 4 days a week, for at least 6 hours a day, during the measurement week. Out of the 4 days, 2 days must be worked from home.
- currently work/reside in the United States/United Kingdom/Hong Kong
- have a good understanding of the English language
- are capable of wearing a wrist-worn sensor
- have continuous access to a smartphone that runs iOs 11+ or Android 7.0+ and know how to use it

## Exclusion criteria

- have no known heart conditions
- are not known to be pregnant
- not have an allergy to silicone
- not have compromised skin (i.e., rashes, wounds) on the wrist
- not have a medical condition that will increase risk of infections due to the electrodes, such as wounds on hand and arm or skin allergies
- not have epilepsy
- not have an active implanted device, such as a pacemaker
- not have a high sensitivity to light or use medication with light-sensitivity side effects.
- not have known nervous system disorders.
- not have a psychiatric diagnosis from a physician or mental health professional
