# Supplementary material for: Stressors and Destressors in Working From Home Based on Context and Physiology From Self-Reports and Smartwatch Measurements: International Observational Study Trial
Source: JMIR Form Res. 2022 Nov 10;6(11):e38562. doi: 10.2196/38562 (PMC9651003; doi:10.2196/38562)
Supplement: Multimedia Appendix 2 [file formative_v6i11e38562_app2.docx]

# Multimedia Appendix 2

## Informed Consent Questions

*These questions will be asked to evaluate whether subjects have understood the protocol. If any of them are answered wrongly, a one-to-one conversation will be scheduled with the participate to discuss the research and answer any questions. It will also evaluate the capability of the subject to join. If the subject seems incapable, he/she will be excluded from the research.*

1. Describe in your own words what the experiment entails:

_______________________

1. How long do you wear the Garmin watch? ___ days
2. When do you wear this watch (as long it’s comfortable)? (circle the right answer)
   1. During workhours only
   2. During workdays only
   3. All 7 days between 9am and 9pm
   4. All day and night
3. How often can you expect the questionnaires during the 7-day measurement period?
   1. Once a day
   2. 3x a day
   3. 5x a day
   4. 12x a day
4. When do you answer the questionnaires?
   1. As fast as possible after the notification has come in
   2. I can answer them all in the evening
   3. Within 4 hours after the questionnaire has become available
   4. Doesn’t matter, I can answer them at any time

## Pre-experiment Questionnaires (prior to 7-day experiment)

### Basic Questions

1. What is your age? ___
2. What is your BMI? ___ (entry is by calculation with length/weight, but only BMI will be stored)
3. Are you male/female/other/do not want to say?
4. What would your job be classified as? manager, scientist, software developer, analyst, customer service, administration, other: ___
5. How many people live in the same house? [0 – 6, more]
6. If >0, how many children are there in the house during a typical week? __ children
7. If >0, what age range are they (multiple answers possible)? [no, 0 years – 25 years, higher]
8. Do you have a dedicated office space at home to minimize distractions? [no, sometimes, yes]
9. Did you work from home pre-COVID times? [no, yes but less, as much as now, more than now]
10. Family and other everyday life events get in the way when working from home [0:not at all – 4:very]
11. I feel like my boss/supervisor/co-workers understands and supports it when work is interrupted by family and other everyday life events during work time [0: not at all – 4: very]
12. I find that I have an intense, demanding job [0:not at all – 4:very]
13. I have a good relationship with my co-workers [0: not at all – 4:very]
14. I find that the pandemic affects my life [0:not at all – 4:very]
15. I have been able to live a full life, despite the pandemic [0:not at all – 4:very]
16. Others (partner, family members, close friends) have helped me deal with everyday life well [0:not at all – 4:very]
17. How does stress normally affects you? (i.e. focus, sleep, social) ____
18. Have you felt burned-out before? Yes in the past /Yes, currently/often/sometimes/Never
19. Do you perform any stress reduction activities? (i.e. mindfulness, meditation, breathing-exercises, yoga) No/Yes, what: ___
20. How anxious are you towards the COVID-19 virus at this moment? [0: not at all – 4:very]
21. Why do you want to participate in the study? ____
22. Do you normally wear a smartwatch? Yes/Sometimes/No

## Daily Questionnaires

### Morning (at 9am)

1. How did you sleep? VAS [bad – good]
2. How stressed did you feel during the night? VAS [not at all – extremely]
3. How stressed do you feel at this moment? VAS [not at all – extremely]
4. Do you expect a stressful day? VAS[not at all – extremely]
5. How tired do you feel at this moment? VAS[not at all – extremely]
6. How happy do you feel at this moment? VAS[not at all – extremely]
7. How motivated do you feel about today? VAS[not at all – extremely]
8. Do you have to take care of others in the house today? ((Home-school) children or elderly/other people) No/Yes, who/what_____
9. Are you working today? Yes/No
10. If yes, where are you working? Home/Office/___
11. If yes, what time do you start and what time do you aim to end? __:__ - __:__, __:__ - __:__ etc.

### Multiple times a day (at 12pm, 3pm, 6pm)

1. How stressed do you feel at this moment? VAS [not at all – extremely]
2. How tired do you feel at this moment? VAS[not at all – extremely]
3. How focused do you feel at this moment? VAS[not at all – extremely]
4. How motivated do you feel at this moment? VAS[not at all – extremely]
5. How productive did you feel over the last 3 hours? VAS[not at all – extremely]
6. At what time did you feel most stressed over the last 3 hours? __:___
7. What caused you stress over the last 3 hours? [work, children, house chores, noise, technical issues, other:___]
8. In the past three hours, how much was present around you of the following:
   1. Sunlight VAS[none - a lot]
   2. Fresh air VAS[none – a lot]
   3. Noise VAS[none - a lot]
   4. If workday, distractions by other people in your house VAS[none – a lot]
   5. If workday, distractions by daily life (i.e. laundry, personal phone calls) VAS[none – a lot]
   6. If non-workday, distractions by work VAS[none – a lot]
9. Have you had social conversations with people over the last 3 hours (not on work topics). No/Yes, with who: colleagues/family/friends/other:___
10. If workday, did you have a break in the last 3 hours? No/Yes, when __:__, __:__,__:__
11. Did you go outside in the last 3 hours? No/Yes, when __:__
12. Did you move actively in the last 3 hours? No/Yes, walking-running-biking-other sports-other: , when: ___:___
13. If non-workday, did you think about work in the last 3 hours? VAS[not at all – a lot]
14. If non-workday, did you do any work? No/Yes, at what times: __:__ - __:__

### Evening (at 9pm)

1. How stressed do you feel at this moment? VAS [not at all – extremely]
2. How tired do you feel at this moment? VAS[not at all – extremely]
3. How focused do you feel at this moment? VAS[not at all – extremely]
4. At what time did you feel most stressed over the last 3 hours? __:___
5. What caused you stress over the last 3 hours? [work, children, house chores, noise, technical issues, other:___]
6. Have you had social conversations with people over the last 3 hours (not on work topics). No/Yes, with who: colleagues/family/friends/other:___
7. Did you go outside in the last 3 hours? No/Yes, when __:__
8. Did you move actively over the last 3 hours? No/Yes, walking-running-biking-other sports-other:____
9. If workday, when did you finish work? __:__
10. If workday, did you do any work after your work-hours? No/Yes, when: __:__ - __:__
11. If workday, did you think about work after your work-hours? VAS[not at all – a lot]
12. If non-workday, did you do any work today? No/Yes, when: __:__
13. If non-workday, did you think about work today? VAS[not at all – a lot]
14. If workday, please fill in any meetings/deadlines you had today and their accompanying stress level (only the times and how much stress you experienced): __:__ stress: VAS[none – a lot]. __:__ stress: VAS[none – a lot].
15. How well were you able to separate your work and personal life today? VAS [not at all – very well]
16. How much was your stress influenced by [0:not at all – 4: a lot]:
    1. Children
    2. House chores
    3. Environmental noise
    4. Technology issues (i.e. laptop failing, wifi-problems)
    5. Work-life separation
    6. Other: _____

## Post-experiment questionnaire

### General opinions

1. How comfortable did the wearable feel? VAS[Uncomfortable – Very comfortable]
2. Would you use a wearable that can tell you when you are stressed? Yes/Maybe/No
3. Would you use a wearable that can tell you when you are stressed and advice on certain things to do to release that stress? Yes/Maybe/No
4. Do you like participating in this kind of research? VAS[not at all – very]
5. Do you think you work more efficiently from home than at location? (without pandemic) No/Sometimes/Equal/Yes
6. Do you find it mentally challenging to work during the pandemic? VAS[not at all – very]
7. Are you more distracted when working from home than at location? No/Sometimes/Equal/Yes
8. What would be your ideal and most-efficient division of working from home vs. working from the office in the future (after the pandemic)? __ days from home, __ days from the office
9. Do you think that the company policy makes it hard to balance work-life balance when working from home?
10. Do you think that the company offers enough flexibility to balance work and life? VAS[not at all – very]
11. What do you find the most difficult about working from home? _______
12. What do you find the most positive about working from home? ________
13. Has participating in this pilot made you more aware of your stress and how it might be impacting you?
14. Did you change anything about your approach to health and well-being as a result of this pilot?
