# Supplementary material for: Stressors and Destressors in Working From Home Based on Context and Physiology From Self-Reports and Smartwatch Measurements: International Observational Study Trial
Source: JMIR Form Res. 2022 Nov 10;6(11):e38562. doi: 10.2196/38562 (PMC9651003; doi:10.2196/38562)

# Multimedia Appendix 3

Figure 1: Stress = “How stressed do you feel at this moment?” on a VAS scale from ‘not at all [0]’ to ‘extremely’ [100], Sunlight, Fresh Air, Noise, Distraction Daily Life, Distraction People = ” In the past three hours, how much was present around you of the following: Sunlight, Fresh Air, Noise, Distractions by daily life, Distractions by other people in the house”. All on a VAS scale from ‘none’[0] to ‘a lot [100]’. An enrichment of the 50 value is visible in all 6 questions.


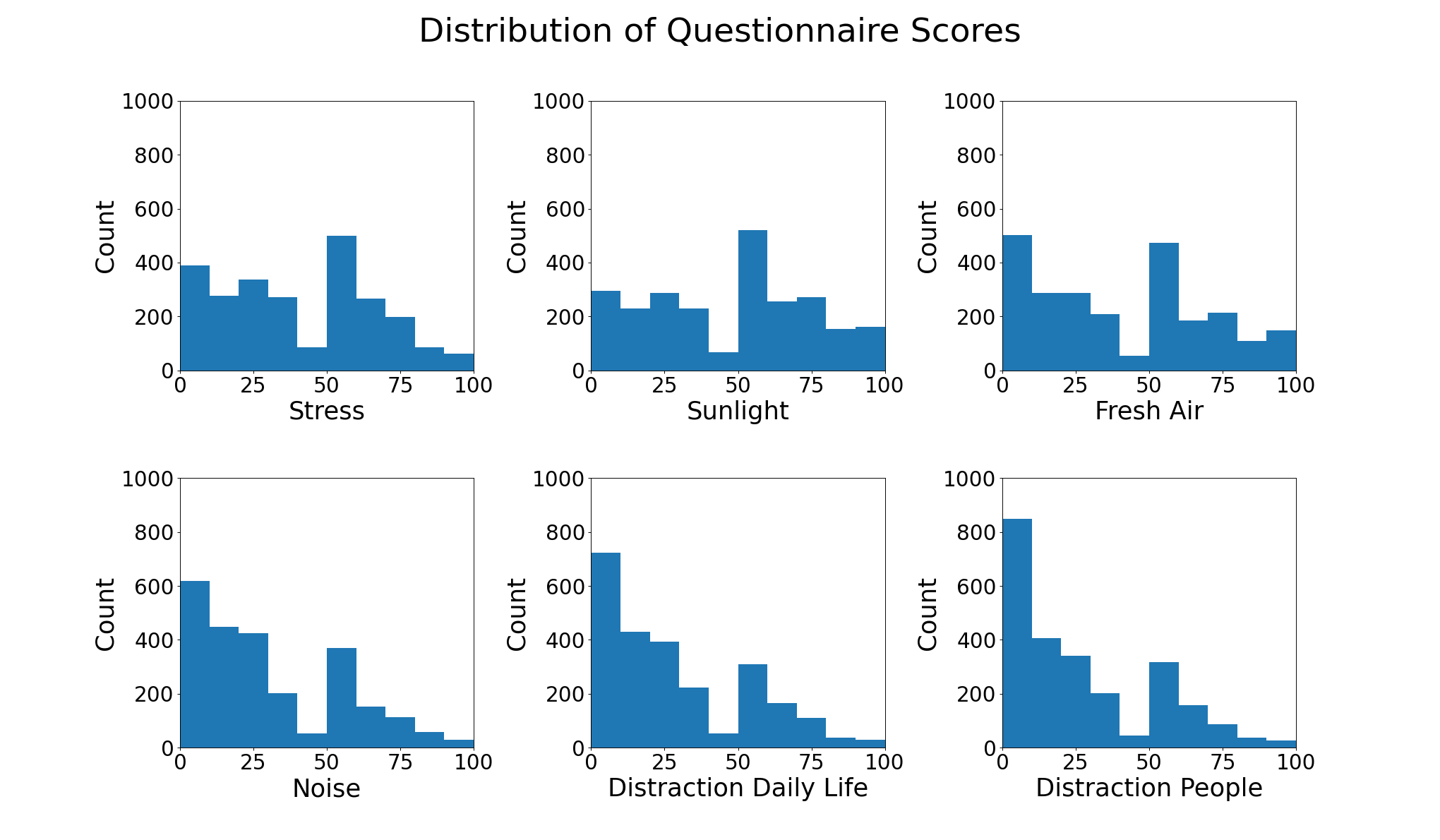

Supplement: Multimedia Appendix 3 [file formative_v6i11e38562_app3.docx]
